# The Obesity Paradox in Patients Undergoing Surgical Repair of Degenerative Mitral Regurgitation

**DOI:** 10.3390/jcm14082817

**Published:** 2025-04-19

**Authors:** Hugo M. N. Issa, Kenza Rahmouni, Alex Nantsios, David Messika-Zeitoun, Marc Ruel, Thierry Mesana, Vincent Chan

**Affiliations:** 1 Division of Cardiac Surgery, University of Ottawa Heart Institute Ottawa, Ottawa, ON K1Y 4W7, Canada; hmonteiro@ottawaheart.ca (H.M.N.I.); krahmouni@ottawaheart.ca (K.R.); anantsios@ottawaheart.ca (A.N.); mruel@ottawaheart.ca (M.R.); tmesana@ottawaheart.ca (T.M.); 2 Division of Cardiology, University of Ottawa Heart Institute Ottawa, Ottawa, ON K1Y 4W7, Canada; dmessika-zeitoun@ottawaheart.ca

**Keywords:** mitral valve, mitral valve repair, survival

## Abstract

**Background:** The obesity paradox describes the beneficial influence of an elevated body mass index on health outcomes. Currently, few studies have evaluated BMI and its impact on survival following surgical repair of degenerative mitral regurgitation (MR). **Methods:** Between 2004 and 2021, 1214 patients underwent surgical mitral valve repair at our institution for MR due to myxomatous degeneration. **Results:** Patient age was 63.2 ± 12.3 years, 341 (28%) were female, and 678 (55%) were either overweight or obese (body mass index ≥ 25 kg/m^2^) preoperatively. Concomitant coronary revascularization was performed in 152 (13%). Clinical and echocardiographic follow-up averaged 4.5 years and was complete for all patients. Perioperative mortality occurred in 4 (0.3%). Ten-year survival, freedom from recurrent MR ≥ 2+, and freedom from recurrent MR ≥ 3+ was 75.1 ± 2.3%, 85.8 ± 2.1%, and 96.6 ± 1.0%, respectively. A higher body mass index was associated with better survival (hazard ratio 0.99, 95% CI 0.98–0.99, *p* = 0.02) after adjusting for age, sex, preoperative LV function, and preoperative LV size. **Conclusions:** A higher BMI was associated with better long-term survival independent of age, gender, LV function, and LV size. These data may provide nuanced risk prognostication in patients undergoing surgical mitral repair.

## 1. Introduction

Obesity is associated with a variety of adverse health outcomes, including an increased risk of developing metabolic syndrome, type 2 diabetes mellitus, and vascular diseases, compared to non-obese individuals [1]. Obesity remains a key public health challenge globally and may be defined by a body mass index (BMI) of ≥30 kg/m^2^ or a body surface area exceeding approximately 2 m^2^ [2]. Governments and health organizations have implemented various public health measures aimed at reducing obesity prevalence, emphasizing preventive strategies such as promoting healthy diets, increasing physical activity, and raising public awareness of the associated risks [3]. The ultimate objective of these interventions is to reduce obesity-related comorbidities and improve overall societal health. However, emerging evidence challenges the uniformly negative view of obesity at a population level, shedding light on a phenomenon known as the “obesity paradox”.

The obesity paradox describes the counterintuitive observation that individuals with obesity may experience better health outcomes in specific medical contexts compared to those with a lower body weight. This paradox has been documented in diverse medical conditions, including cancer and osteoporosis, where obese patients demonstrate more favorable outcomes in certain scenarios [4,5]. Within the realm of cardiovascular medicine, the obesity paradox is particularly well-documented. Notably, patients with heart failure who are classified as overweight or obese exhibit significantly improved survival rates compared to individuals of normal weight. For instance, Horwich et al. reported that obese and overweight patients with moderate-to-severe heart failure achieved better 5-year survival than their normal-weight counterparts [6]. This finding was further substantiated by a recent meta-analysis encompassing nine studies, which demonstrated improved overall survival and reduced cardiovascular mortality among overweight and obese individuals relative to those of normal weight [7].

The obesity paradox also extends to patients undergoing cardiac interventions, including coronary revascularization and transcatheter aortic valve implantation (TAVI). A meta-analysis revealed that overweight and obese patients experienced lower overall and cardiovascular mortality following coronary revascularization compared to individuals of a lower weight [8]. Similarly, despite a higher prevalence of comorbidities and an increased incidence of vascular complications during TAVI procedures, obese patients had better 1-year survival rates compared to normal-weight individuals [9]. Interestingly, this survival advantage appears to be consistent across diverse patient populations undergoing TAVI in Asia and Europe, highlighting the reproducibility of this phenomenon across different healthcare systems and demographic cohorts [10,11,12].

While the obesity paradox has been extensively explored in the context of aortic valve replacement, there is a relative paucity of data examining its impact on outcomes following other types of valve surgery. For example, degenerative mitral regurgitation (MR) represents a clinically significant condition, given the prevalence of mitral valve prolapse within the general population [13,14,15,16]. Patients with degenerative MR undergoing surgical intervention constitute a particularly important subgroup for investigation due to the unique anatomical and physiological challenges associated with this condition.

To address this gap in the knowledge, we conducted a comprehensive cohort study involving 1214 patients who underwent surgical repair of degenerative MR. Our investigation focused on assessing the influence of BMI on key outcomes, including overall survival and freedom from recurrent MR. Understanding the interplay between BMI and surgical outcomes in this context may be important for refining patient selection, tailoring perioperative management strategies, and ultimately improving long-term prognosis. This research contributes to the growing body of evidence on the obesity paradox, with implications that extend beyond aortic valve replacement to encompass other areas of cardiac and non-cardiac surgery. Further exploration of this phenomenon is warranted to elucidate the underlying mechanisms and to refine clinical decision-making in the management of obese patients.

## 2. Patients and Methods

### 2.1. Ethics Approval and Funding

This study has ethics approval from our institutional research ethics board at the University of Ottawa Heart Institute to evaluate patients (Protocol 20160395-01H). Individual consent was waived. This work has not received funding.

### 2.2. Patient Population and Surgical Technique

Between 2004 and 2021, 1214 patients underwent surgical correction of degenerative MR at the University of Ottawa Heart Institute. Patients were excluded if they had functional MR or mixed mitral valve disease with regurgitant and stenotic components.

Patients underwent mitral repair via median sternotomy or mini-thoracotomy, with cardiopulmonary bypass and cardioplegic arrest. Patients underwent mitral annuloplasty with the Medtronic Futureband (Minneapolis, MN, USA) in 1068 cases (88%), Edwards Physio (Irvine, CA, USA) in 76 cases (6%), the Medtronic Duran band (Minneapolis, MN, USA) in 67 cases (6%), and the Cosgrove band in 3 cases (0.2%).

Mitral leaflet repair included resection in 371 patients (31%), neochordal replacement in 383 patients (32%), and chordal transfer in 209 patients (17%). Baseline patient characteristics are described in Table 1.

### 2.3. Echocardiographic Assessment and Follow-Up

All patients had a pre-operative transthoracic and/or transesophageal echocardiogram to measure cardiac dimensions, transvalvular pressure gradients, biventricular function, and valve regurgitation severity, as recommended by the American Society of Echocardiography [17,18]. Patients were prospectively followed at the University of Ottawa, which has previously been described [13,14,15,16] and includes a combination of in-person clinic visits and telephone interviews. Patients were assessed at discrete intervals over the course of the follow-up [16].

Echocardiographic follow-up was complete for all patients, with a total of 4002 echocardiographic assessments for these 1214 patients with an average follow-up assessment interval of 4.5 ± 3.7 years.

### 2.4. Surgical Technique

All patients in the study underwent mitral valve repair via a median sternotomy or mini-thoracotomy, with the use of cardiopulmonary bypass and cardioplegic arrest. Annuloplasty was performed for all patients using the Medtronic Futureband (Minneapolis, MN, USA) in 341 cases, Carpentier Edwards Physio ring (Irvine, CA, USA) in 21 cases, or the Medtronic Duran band (Minneapolis, MN, USA) in 2 cases, considering the intertrigonal distance and the anterior mitral valve leaflet. All mitral repair procedures were performed by two surgeons. Adjunctive techniques included placement of an artificial neochordae in 383 patients (32%), resection in 371 patients (31%), and chordal transfer in 209 patients (17%). Concomitant coronary revascularization was performed in 152 patients (13%), tricuspid valve repair in 174 patients (14%), and aortic valve replacement in 42 patients (3%).

### 2.5. Statistical Analysis

Data were imported and analyzed with Stata data analysis and statistical software version 15.1 (StatCorp, College Station, TX, USA). Continuous variables were described as mean ± standard deviation, and count data were reported as discrete values and a proportion of the total. Survival, freedom from recurrent MR ≥ 2+, and freedom from recurrent MR ≥ 3+ were assessed using the Kaplan–Meier method. A Cox proportional hazards model was used to assess risk factors associated with mortality.

## 3. Results

### 3.1. Preoperative and Operative Characteristics

Table 1 outlines the pre-operative and operative characteristics of patients following surgical mitral valve repair. Patients in this cohort were of a mean age of 63.2 ± 12.3 years and predominantly (92%) had preserved LV function. Three hundred forty-one (28%) were female, and the average body surface area of patients in this study was 1.99 ± 0.18 m^2^. The mean RVSP was 40.9 ± 14.4 mmHg. The mean BMI in this population was 26.0 ± 4.6 kg/m^2^, and 678 (56%) were either overweight or obese with a BMI ≥ 25 kg/m^2^.

### 3.2. Survival

**Figure 1:**
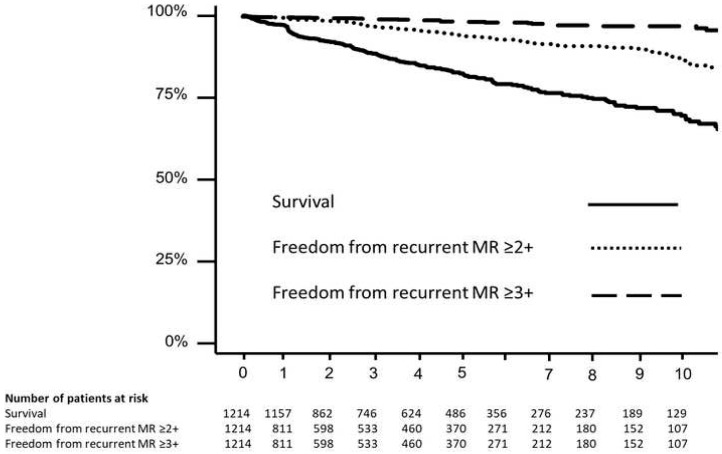
Outcomes after surgical mitral valve repair. Survival (black line), freedom from recurrent mitral regurgitation ≥ 2+ (black dotted line), and freedom from recurrent MR ≥ 3+ (black dashed line) are described.

There were four perioperative deaths (0.3%). Over the course of the study follow-up, there were 171 deaths. Ten-year survival was 75.1 ± 2.3% (Figure 1). Age and BMI were independently associated with survival after adjusting for sex and preoperative LV size and function (Table 2).

### 3.3. Mitral Regurgitation Recurrence

Over the course of the follow-up, 68 patients developed MR ≥ 2+, of which 17 had recurrent MR ≥ 3+. Subsequent mitral valve reoperation was performed in five. Ten-year freedom from recurrent MR ≥ 2+ and recurrent MR ≥ 3+ was 85.8 ± 2.1% and 96.6 ± 1.0%, respectively (Figure 1). Notably, BMI was not associated with the development of recurrent MR ≥ 2+ (HR 1.02, 95% confidence interval 0.97–1.08, *p* = 0.4).

## 4. Comment

In this study, we assessed the relationship between body mass index (BMI) and postoperative outcomes following the surgical repair of degenerative mitral regurgitation (MR). Our analysis indicates an association between higher BMI and enhanced survival, a relationship that persists independently of key factors such as age, female sex, preoperative left ventricular (LV) function, and preoperative LV size. These findings provide novel insights into how BMI influences outcome and long-term prognosis in patients undergoing surgical intervention for degenerative MR.

Remarkably, over half of the patients in our cohort were classified as either overweight or obese, highlighting the evolving demographic trends in BMI distributions worldwide. The proportion of obese individuals within our study cohort was 15%, which is notably lower than the approximately 29% obesity prevalence reported among Canadians in 2016 [19]. Additionally, the patient population in this cohort predominantly had preserved LV function, thereby representing a relatively healthy patient group at the time of surgery. This specific demographic profile likely played an important role in the favorable outcomes of the overall cohort, although the impact of BMI on outcomes in this present study persisted after adjusting for LV function.

Outcomes for this cohort were favorable, with perioperative mortality of 0.3%, even when considering that approximately 20% of patients in this cohort underwent a concomitant surgical procedure. This low perioperative mortality underscores the durability and safety of surgical interventions in the context of degenerative MR, aligning favorably with previous studies in the field [20]. Furthermore, long-term survival outcomes also demonstrated consistency with the existing literature, underscoring the efficacy of mitral valve repair as a durable solution [21]. The durability of the surgical repairs was highlighted by the low incidence of recurrent MR across the follow-up period, reaffirming the utility of surgery as a robust treatment option.

### 4.1. Obesity Paradox in the Literature

The finding that higher BMI positively correlates with improved survival is particularly intriguing when contextualized within the broader body of literature on valvular heart disease. This so-called “obesity paradox”, observed in other interventions such as transcatheter aortic valve implantation (TAVI), provides a parallel framework for understanding the complex interplay between body composition and postoperative outcomes. Notably, Om et al. reported a survival benefit at 1-year follow-up for patients with a BMI ≥ 24.9 kg/m^2^ undergoing TAVI [10], while Gonzalez-Ferreiro et al. highlighted superior 3-year survival outcomes among obese patients with a BMI ≥ 30 kg/m^2^ compared to those with lower BMI levels [11]. Despite these insights, research specifically evaluating the impact of BMI in the context of surgical mitral repair remains limited.

### 4.2. What Is the Mechanism of the Obesity Paradox?

From a mechanistic perspective, the association between higher BMI and improved survival warrants further investigation. Potential explanations may include a greater metabolic reserve in patients with higher BMI, which could provide resilience against surgical stress and postoperative recovery challenges. Indeed, metabolic syndrome and insulin resistance may not be observed in all obese patients. Meigs et al., in a population study involving 2902 individuals, found that metabolic syndrome was observed in 37% of obese patients, whereas 7% of normal weight individuals had metabolic syndrome [22]. Obese patients with metabolic syndrome had a 10-fold risk of developing diabetes mellitus [22]. Indeed, others have also shown that obesity and insulin resistance combined may not portend the development of ischemic heart disease [23].

The distribution of fat may also have an impact on clinical outcomes. Indeed, visceral fat deposition along with a relative lack of lean muscle has been implicated in the development of adverse cardiovascular events [24,25].

Additionally, hormonal and inflammatory mediators unique to individuals with higher BMI may also contribute to improved outcomes, though these hypotheses require future exploration to validate. The published literature does support the notion that obesity is associated with an increase in many inflammatory markers. Obese patients may have fewer anti-inflammatory M2 macrophages compared with pro-inflammatory M1 macrophages, which was also correlated with insulin resistance [26]. Obese individuals have also been found to have higher circulating levels of pro-inflammatory hormones such as leptin, visfatin, and resistin [26].

### 4.3. Clinical Implications

The increase in obesity prevalence in the general population may also lead to more obese patients presenting for cardiac surgery. Research in this field advocates reducing perioperative risk via facilitating early extubation and mitigation of cardiopulmonary bypass if possible [27]. In addition, sternal fixation devices may also be employed to further stabilize the thoracic cage [28].

### 4.4. Future Directions

Future studies should aim to elucidate the underlying mechanisms driving this observed association and explore whether tailored preoperative or postoperative strategies based on BMI can further optimize patient care. By advancing our understanding of these dynamics, we can continue to improve decision-making processes, refine surgical techniques, and ultimately enhance the long-term prognosis for patients with degenerative MR. This work reinforces the critical importance of considering patient-specific factors, such as BMI, to achieve personalized and effective treatment outcomes in mitral valve surgery.

### 4.5. Limitations

This was a non-randomized cohort study involving prospectively collected data; therefore, there may be unknown confounders that could introduce bias. The number of patients on the extreme ends of the body mass index was small, which precluded the determination of a dose–response effect with obesity. Specifically, only 15% of patients in this cohort were obese. Data were analyzed as a continuous variable, but having more patients in the obese category may have allowed for different analyses, which may have led to the identification of a more nuanced impact of BMI on outcomes (perhaps a J-shaped curve) that was not demonstrated in this study.

## 5. Conclusions

In conclusion, this study offers evidence supporting the efficacy and durability of surgical repair for degenerative MR, particularly in a cohort comprising overweight or obese individuals with preserved LV function. This study provides novel evidence describing the inverse relationship between death and body mass index following surgical repair of degenerative mitral valve regurgitation. These findings not only add to the existing literature on valvular heart disease but also open avenues for further research into the nuanced role of BMI in shaping surgical outcomes.

## Figures and Tables

**Table 1:** Patient baseline and operative characteristics.

Variable	Value
Age (years)	63.2 ± 12.3
Body mass index (kg/m^2^) BMI ≥ 30	187 (15%)
25 ≤ BMI < 30	491 (40%)
18.5 ≤ BMI < 25	501 (41%)
BMI < 18.5	35 (3%)
Body surface area (m^2^)	1.91 ± 0.22
Female sex	341 (28%)
Echocardiographic details	
Anterior leaflet prolapse	578 (48%)
Bileaflet prolapse	207 (17%)
Posterior leaflet prolapse	1015 (84%)
Indexed LVESD (mm/m^2^)	19.0 ± 4.5 mm/m^2^
LVEDD (mm)	56.7 ± 14.2
LVESD (mm)	39.6 ± 10.4
LVEF (%)	55.2 ± 8.2
RVSP (mmHg)	40.9 ± 14.4
MAC	278 (23%)
Surgical Technique	
Aortic valve replacement	42 (3%)
CABG	152 (13%)
Maze procedure	242 (20%)
Mitral valve repair technique	
Chordal transfer	209 (17%)
Neo chordal replacement	383 (32%)
Resection	371 (31%)
Tricuspid valve repair	174 (14%)

BMI, Body mass index; CABG, coronary artery bypass grafting; LV, left ventricle; LVEDD, left ventricular end-diastolic diameter; LVEF, left ventricular ejection fraction; LVESD, left ventricular end-systolic diameter; MAC, mitral annular calcification; RVSP, right ventricular systolic pressure.

**Table 2:** Risk factors associated with mortality.

Covariate	Hazard Ratio (95% Confidence Interval)	*p*-Value
Age	1.07 (1.05–1.08)	<0.001
Body mass index	0.99 (0.98–0.99)	0.02
Female sex	0.83 (0.58–1.18)	0.3
Preoperative LV end-systolic dimension	1.00 (0.98–1.02)	0.8
Preoperative LV class *	1.60 (1.37–1.87)	<0.001

LV, Left ventricle. * LV grade 1 = LVEF ≥ 60%; grade II = LVEF ≥ 35% and <60%; grade III = LVEF ≥ 20% and <35%; grade IV = LVEF < 20%.

## Data Availability

Data may be available upon reasonable request.
